# Relapsing Chronic Immune Thrombocytopenia and Thrombosis: A Case Report

**DOI:** 10.7759/cureus.91795

**Published:** 2025-09-07

**Authors:** Lara Kose

**Affiliations:** 1 Department of Medicine, University of California Los Angeles David Geffen School of Medicine, Los Angeles, USA

**Keywords:** antiphospholipid antibody, diagnosis and management of thrombocytopenia, immune thrombocytopenia, platelet disorder, refractory thrombotic thrombocytopenic purpura, thrombocytopenia

## Abstract

Immune thrombocytopenia (ITP) involves an acquired, immune-mediated disorder involving autoantibodies, which target platelet antigens. It may be primary or secondary to an underlying condition. While some may be asymptomatic, others may experience easy bruising, petechiae, purpura, epistaxis, bleeding from the gums, menorrhagia, hematuria, melena, or hematochezia. This is a case of a 21-year-old female who presented to the Emergency Room after experiencing several days of intermittent bruising and menorrhagia. She was found to have a platelet count of 16,000/μL and hemoglobin of 9.7 g/dL with a normal white blood cell count. One of the many clinical challenges for ITP corresponds to the lack of sensitive or specific diagnostic tests. Treatment is strongly advised for those with active bleeding, severe thrombocytopenia or if an invasive procedure is needed. Options for treatment are initially limited to corticosteroids. Alternative treatment options include intravenous immunoglobulin, rituximab, and thrombopoietic receptor agonists. This case was complicated by a recurrence of thrombocytopenia with bleeding despite corticosteroid treatment, followed by thrombosis in the setting of possible antiphospholipid syndrome. The goals for treatment involved not only increasing the platelet count to a level that decreases the risk of bleeding complications but also improving the patient's quality of life. Furthermore, clinical challenges were involved when considering the management of thrombosis in a patient with chronic ITP.

## Introduction

Immune thrombocytopenia (ITP) involves an acquired autoimmune thrombocytopenia caused by platelet membrane antigens (glycoproteins) targeted by autoantibodies. Thrombocytopenia is defined as a platelet count below the reference range, typically under 100,000/μL for adults [1]. The reference range for a normal platelet count in adults is 143-398/μL It may be primary or secondary to an underlying condition such as an autoimmune disease or hematologic malignancy. The incidence of ITP is estimated to be two to five per 100,000 persons in the general population [2]. Diagnostic challenges encompass working through the differential diagnosis that is composed of other causes of thrombocytopenia, including thrombotic microangiopathy syndrome, which can present with microangiopathic hemolytic anemia (MAHA) and thrombocytopenia [3]. Evans syndrome can involve ITP in the presence of warm autoimmune hemolytic anemia. Currently, there are no sensitive or specific diagnostic tests for ITP. Primary ITP, in which an underlying etiology is not identified, can be considered a diagnosis of exclusion. On the other hand, secondary ITP is associated with various chronic conditions including, but not limited to, autoimmune disease (systemic lupus erythematosus, antiphospholipid syndrome, rheumatoid arthritis), hematologic malignancies (chronic lymphocytic leukemia, lymphoma), and chronic infectious diseases (human immunodeficiency virus, hepatitis C virus, cytomegalovirus, *Helicobacter pylori*) [1]. While some may be asymptomatic, others may experience easy bruising, petechiae, purpura, epistaxis, bleeding from the gums, menorrhagia, hematuria, melena, or hematochezia. An extensive diagnostic evaluation is highly valuable as it can determine how to proceed with treatment, which varies depending on the underlying etiology.

## Case presentation

A 21-year-old female, with a history of prediabetes and hyperlipidemia, presented to the Emergency Room after experiencing several days of intermittent bruising and menorrhagia. She was found to have a platelet count of 16,000/μL (normal reference range: 143-398/μL) and a hemoglobin level of 9.7 g/dL (normal reference range: 11.6-15.2 g/dL), as noted in Table 1, which includes the results from the lab testing on initial presentation.

**Table 1:** Results from initial laboratory testing are noted, with findings significant for thrombocytopenia and anemia.

Laboratory testing	Results	Reference range	Value interpretation
Platelet	27,000/μL	143,000-398,000/μL	Low
White blood cell count	5,800/μL	4,000-11,000/μL	Normal
Hemoglobin	10.4 g/dL	11.7-15.7 g/dL	Low
Hematocrit	31.7%	37.0-47.0%	Low
Mean corpuscular volume	83 fL	82-98 fL	Normal
Mean corpuscular hemoglobin	27.2 pg	27.0-34.0 pg	Normal
Mean corpuscular hemoglobin concentration	32.8 g/dL	32.0-36.0 g/dL	Normal
Red cell distribution width	13.9%	11.5-14.5%	Normal
Prothrombin time	13.3 seconds	9.8-12.8 seconds	Elevated
Partial thromboplastin time	51 seconds	25-38 seconds	Elevated
Sodium	142 mmol/L	136-145 mmol/L	Normal
Potassium	3.6 mmol/L	3.6-5.1 mmol/L	Normal
Chloride	109 mmol/L	98-107 mmol/L	Normal
Carbon dioxide	25 mmol/L	21-32 mmol/L	Normal
Calcium	8.9 mg/dL	8.5-10.1 mg/dL	Normal
Glucose	107 mg/dL	74-99 mg/dL	Mildly elevated
Creatinine	0.70 mg/dL	0.55-1.02 mg/dL	Normal
Total bilirubin	0.3 mg/dL	0.2-1.0 mg/dL	Normal
Alanine aminotransferase	14 IU/L	13-56 1U/L	Normal
Aspartate aminotransferase	12 IU/L	10-37 IU/L	Normal
Alkaline phosphatase	69 IU/L	45-117 IU/L	Normal

After receiving 1 unit of platelets, her platelet count increased, followed by a decline 12 hours later. She received prednisone 80 mg once daily for a few days until outpatient follow-up after hospital discharge. Additional testing revealed that she was Coombs positive. During this time, hemoglobin levels gradually improved to the normal range with a daily iron supplement. Since then, she had been following up with a hematologist. A steroid taper was followed by three relapsing courses of thrombocytopenia, which occurred during menstrual periods with severe menorrhagia. She was also developing petechiae in her mouth and legs. She remained on prednisone daily with doses ranging from 10 mg daily to 60 mg daily, depending on treatment response. The dose was increased after presenting with bleeding blisters in the mouth, epistaxis, and petechiae in her legs. This was followed by symptom resolution, after which the prednisone taper was initiated. Rituximab treatment was then initiated to ensure that the patient could remain off prednisone. After four weekly infusions of rituximab, her platelet count normalized, and she was transitioned off prednisone. Table 2 provides the trend in platelet counts throughout the course of the patient’s treatment.

**Table 2:** Trends in platelet counts over the course of treatment. The platelet counts improve while on prednisone but decline while weaning down to lower doses. This coincides with a recurrence of symptoms, including petechiae of the mouth and legs. With higher doses of prednisone and improvement of platelet counts, the patient's symptoms improved. Rituximab was later initiated to ensure that the prednisone could be tapered down while maintaining normal platelet counts.

Treatment course	Platelet count (/μL) (reference range: 143,000-398,000/μL)
Initial presentation prior to treatment	16,000
1 unit of platelets received	39,000
12 hours post platelet transfusion	4,000
Prednisone 80 mg daily initiated	255,000
Tapered off prednisone	2,000
Prednisone 60 mg daily	20,000
Prednisone 40 mg daily	220,000
Prednisone 20 mg daily	75,000
Prednisone 40 mg daily	234,000
Prednisone 20 mg daily	1,000
Prednisone 80 mg daily	33,000
Prednisone 80 mg daily	531,000
Prednisone 40 mg daily	284,000
Prednisone 10 mg daily with 1st dose of rituximab	260,000
Prednisone 5 mg daily	250,000
Prednisone taper and fourth dose of rituximab completed	286,000

Two years later, the patient was found to have a deep vein thrombosis (DVT) in the left posterior tibial and peroneal veins. At the time, she was on an estrogen-containing oral contraceptive but otherwise had no other provoking factors. Her platelet count had been within the normal range. She was started on a six-month course of apixaban. The oral contraceptive was replaced by a progesterone-only contraceptive, which controlled the menorrhagia. The following year, she was found to have a DVT in the right femoral, accessory femoral, popliteal, and peritoneal veins. The patient's platelet count was 142,000/μL, and she did not have any symptoms involving bleeding or bruising. Since she was not on any anticoagulation at the time, apixaban was restarted. Thrombophilia testing was significant for elevated cardiolipin IgM and IgG levels and a positive dilute Russell viper venom time on two separate occasions at least 12 weeks apart, suggesting the possibility of antiphospholipid syndrome. Table 3 lists the results from the thrombophilia lab testing. Genetic testing did not reveal any significant findings. Additional lab testing was done by Rheumatology to assess the potential for an underlying autoimmune condition (Table 4). The results were notable for an elevated anti-nuclear antibody titer of 1:160 and a mildly elevated C-reactive protein of 0.9 mg/dL. However, with the remaining normal test results, the patient did not meet criteria for autoimmune conditions, such as systemic lupus erythematosus or rheumatoid arthritis.

**Table 3:** Lab test results to assess thrombophilia. Tests used to assess thrombophilia after the patient was diagnosed with her second DVT were significant for elevated cardiolipin IgM and IgG along with a positive dilute Russell viper venom time. For confirmatory testing, these tests were repeated approximately 12 weeks later. The results suggest the possibility of antiphospholipid syndrome. The protein C clottable activity was only minimally elevated.

Laboratory testing	Results from initial testing	Results 12 weeks after initial testing	Reference range	Value interpretation
Protein C, clottable activity	132%	-	70-130%	Minimally elevated
Protein S, functional activity	76%	-	57-131%	Normal
Antithrombin III antigen	102%	-	82-136%	Normal
Cardiolipin IgG	83.5 CU	89.9 CU	≤20.0 CU	Elevated
Cardiolipin IgM	115.9 CU	105.4 CU	≤20.0 CU	Elevated
Cardiolipin IgA	<20.0 CU	<20.0 CU	≤20.0 CU	Normal
dilute Russell viper venom time	Positive	Positive	Negative	Positive

**Table 4:** Lab test results to evaluate for a potential underlying autoimmune etiology. Lab tests to evaluate for a potential underlying autoimmune etiology reveal non-specific elevations in anti-nuclear antibody and C-reactive protein levels. C-ANCA, cytoplasmic anti-neutrophil cytoplasmic antibody; P-ANCA, perinuclear anti-neutrophil cytoplasmic antibody; SSA, Sjögren’s-syndrome-related antigen A; SSB, Sjögren’s-syndrome-related antigen B; Sm, anti-Smith; RNP, anti-ribonucleoprotein; dsDNA, double-stranded DNA

Laboratory testing	Results	Reference range	Value interpretation
Anti-nuclear antibody	1:160 titer, homogeneous and speckled	<1:40 titer	Elevated
Neutrophil cytoplasmic antibody, C-ANCA	<1:20	<1:20 titer	Normal
Neutrophil cytoplasmic antibody, P-ANCA	<1:20	<1:20 titer	Normal
Neutrophil cytoplasmic antibody, proteinase 3 antibody	<20.0 CU	<20.0 CU	Normal
Myeloperoxidase antibody	<20.0 CU	<20.0 CU	Normal
SSA antibody	<20.0 U	<20.0 U	Normal
SSB antibody	<20.0 U	<20.0 U	Normal
Sm antibody	<20.0 U	<20.0 U	Normal
RNP antibody	<20.0 U	<20.0 U	Normal
dsDNA antibody	≤200 IU/mL	≤200 IU/mL	Normal
C3 complement	169 mg/dL	86-175 mg/dL	Normal
C4 complement	34 mg/dL	10-40 mg/dL	Normal
Rheumatoid factor	10 IU/mL	<14 IU/mL	Normal
Cyclic citrullinated peptide antibody	4 units	0-19 units	Normal
Erythrocyte sedimentation rate	10 mm/hr	<25 mm/hr	Normal
C-reactive protein	0.9 mg/dL	<0.8 mg/dL	Mildly elevated
Thyroid peroxidase antibody	18 IU/mL	≤20 IU/mL	Normal

The patient was monitored closely while she remained on long-term anticoagulation. She did not have any bleeding complications, and her platelet counts remained within the normal range. Overall, the case proved to be challenging given the recurrent bleeding in the setting of refractory thrombocytopenia. However, the thrombotic events indicate the conflicting nature of ITP in this case, which prompted further investigation of secondary ITP.

## Discussion

ITP is known to be an acquired bleeding disorder most commonly seen in adults. While the pathogenesis of primary ITP is not entirely understood, it is thought to involve specific autoantibodies, such as IgG, which target platelet membrane glycoproteins. The outcome may involve immune-mediated platelet destruction and/or suppressed platelet production [4]. The anti-glycoprotein autoantibodies attached to platelets are destroyed by macrophages via Fcg receptors in the reticuloendothelial system. Platelet destruction may also be accelerated by autoantibodies using complement-dependent cytotoxicity and platelet apoptosis, which can take place in the blood, spleen, and liver. The pathogenesis may also involve inappropriate bone marrow production of platelets due to an autoimmune response against megakaryocytes with glycoproteins that are similar to that seen on platelets. They are then recognized by complement-dependent cytotoxicity and antibody-dependent cellular phagocytosis. The bone marrow may also be the site in which cytotoxic T cells can target platelets [5]. The heterogeneity of this condition can be attributed to the multiple potential pathophysiologic mechanisms involved. It can be further classified as acute or newly diagnosed if the duration of illness is under three months, persistent if between 3 and 12 months and chronic if over 12 months [5,6].

ITP remains a diagnosis of exclusion due to the lack of a “gold standard” diagnostic test. When first encountering thrombocytopenia, a series of steps can be taken to determine how to proceed with management. The urgency of the case can be determined by the level of thrombocytopenia and the presence of complications including bleeding and thrombosis. Repeating the complete blood count to ensure a laboratory error can be the first step. Additional studies to consider may include the peripheral blood smear to evaluate for artifact due to platelet clumping, abnormalities in platelet morphology, and the presence of schistocytes. The latter would direct one to question hemolysis, for which a complete blood count panel, reticulocyte panel to assess bone marrow activity, serum haptoglobin level, serum lactate dehydrogenase level, direct antiglobulin test, indirect bilirubin, serum creatinine, coagulation studies (including prothrombin time, activated partial thromboplastin time, serum fibrinogen level), and an ADAMTS13 activity and inhibitor level would be beneficial. Thrombotic thrombocytopenic purpura (TTP) involves the presence of severe thrombocytopenia and MAHA. Biallelic pathogenic mutations in the *ADAMTS13* gene will confirm the diagnosis [3]. The Chinese ITP guidelines recommend bone marrow aspiration smear with biopsy, flow cytometry, and cytogenetic analysis if there is concern for aplastic anemia or myelodysplastic syndrome. This can also be helpful when suspecting leukemia or lymphoma. While an anti-glycoprotein autoantibody level is highly specific for diagnosing ITP, the sensitivity remains low [6,7].

With the positive Coombs test result in the setting of significantly low platelet counts and anemia, Evans syndrome can be considered. It is known as a rare autoimmune disease involving at least two of the following autoimmune cytopenias: ITP, autoimmune hemolytic anemia (with a positive direct Coombs test, hemoglobin less than 12 g/dL), autoimmune neutropenia. After the second incident of autoimmune cytopenia, the diagnosis of Evans syndrome can be met [8]. Thus far, the diagnostic findings in this patient's case may suggest the presence of autoimmune hemolytic anemia in the setting of thrombocytopenia. However, the patient's anemia could be attributed to blood loss from menorrhagia, which later improved with a daily iron supplement.

Causes of secondary ITP in adults account for approximately 20% of cases and include a number of chronic disorders and infectious diseases including, but not limited to, chronic lymphocytic leukemia, systemic lupus erythematosus, antiphospholipid syndrome, *Helicobacter pylori* infection, HIV infection, hepatitis C infection, cytomegalovirus infection, varicella zoster virus infection, and Coronavirus disease (COVID-19). There are also cases of drug-induced thrombocytopenia, such as that seen with antibiotics, including sulfonamides, ampicillin, piperacillin, and vancomycin [1]. Determining the distinction between primary and secondary ITP can provide insight into other potential comorbidities. For instance, in this particular case, the patient had developed DVT. While the initial DVT in the left lower extremity was thought to be potentially provoked by the use of an estrogen-containing oral contraceptive, the development of the right lower extremity DVT led to questioning the possibility of thrombophilia and other predisposing factors.

Antiphospholipid syndrome is an autoimmune condition and a common type of acquired thrombophilia. Diagnostic criteria is met in the presence of antiphospholipid antibodies (including anticardiolipin antibodies, anti-beta2-glycoprotein I) and positive lupus anticoagulant testing (dilute Russell viper venom time) in the setting of at least one clinical manifestation, such as venous or arterial thrombosis or adverse pregnancy outcomes (at least one unexplained death of a fetus at least 10 weeks of gestation, at least one premature delivery of a fetus less than 34 weeks of gestation in the setting of severe preeclampsia or eclampsia, at least three unexplained consecutive miscarriages at less than 10 weeks of gestation). Thrombocytopenia is commonly associated with this condition; however, it is not part of the diagnostic criteria. It may also be common to encounter thrombocytopenia and antiphospholipid antibodies while not meeting the clinical criteria for antiphospholipid syndrome. The presence of thrombocytopenia is not expected to provide protection against the increased thrombotic risk [9]. Therefore, anticoagulation is indicated. Caution is advised with the use of anticoagulation and the increased bleeding risk associated with thrombocytopenia. For women of child-bearing age, a discussion with shared decision-making is needed regarding menstrual management, contraception choice, and family planning.

According to the American Society of Hematology guideline panel, treatment with corticosteroids is recommended for newly diagnosed ITP and a platelet count of less than 30,000/μL and for those who are asymptomatic or have minor mucocutaneous bleeding. If the platelet count is under 20,000/μL, hospital admission is recommended. Prednisone can be given over a four- to right-week course with a starting dose of 0.5-2mg/kg daily or dexamethasone with a pulse dose of 40 mg daily for four days for up to three four-day courses. Improvements in platelet counts may be seen two to four days after starting treatment. However, 70% of patients may relapse and require second-line therapy as long-term steroid use is not advised due to a higher risk of complications or adverse effects. A widely used option for refractory ITP includes, rituximab, which is an anti-CD20+ B cell monoclonal antibody. While it is known to achieve long remissions after a single treatment course, it may involve a relapse after 1.5 years. Potential adverse effects include infusion reactions, increased risk of infections, hypogammaglobulinemia, and neutropenia. For those with hepatitis B viral infection, there is a risk of viral reactivation [9]. The PROLONG trial is currently investigating rituximab as maintenance therapy in ITP [10]. Therapeutic plasma exchange is the treatment of choice specifically for TTP [3].

Alternative treatment options for ITP include intravenous immunoglobulins (IVIg), thrombopoietin receptor agonists (TPO-RAs), and splenic tyrosine kinase inhibitors [2,9]. While IVIg can lead to an increase in platelet counts in more than 80% of newly diagnosed ITP patients, it is expensive and the response rate is typically transient. Caution will need to be considered for those with impaired renal function and an increased risk of thrombosis [6]. TPO-RAs (romiplostim, eltrombopag, avatrombopag) have been commonly used treatments for relapsed/refractory ITP. Their mechanism of action involves stimulating platelet production by binding to c-mpl, the human thrombopoietin receptor. While there are few randomized studies confirming their efficacy, thus far romiplostim has achieved an efficacy range of 74-96%. Fostamatinib, a splenic tyrosine kinase inhibitor, reduces antibody-mediated platelet destruction with the inhibition of signaling at the B-lymphocyte receptors and Fc-activating receptors. In a clinical trial, it achieved an efficacy of 43%. Refractory ITP has also been treated with the following: azathioprine, cyclophosphamide, cyclosporin A, danazol, dapsone, mycophenolate mofetil, vinblastine, and vincristine. Lastly, splenectomy may be indicated in refractory cases with the intent to remove the site of platelet destruction [9]. In this case report, treatment success was achieved with the use of prednisone followed by rituximab.

## Conclusions

ITP can potentially be a challenging transient or chronic condition to treat. As its pathogenesis continues to be an area of study, corticosteroids remain the first-line treatment. However, this option would be preferred if a short course of treatment is sufficient. In refractory cases, alternative treatment options will need to be considered. The case presented offers a glimpse of the complicated nature of thrombocytopenia with not only management but also secondary causes that can conflict with the treatment provided. With the possibility of antiphospholipid syndrome in the setting of recurrent venous thrombosis, anticoagulation is approached with caution while closely monitoring platelet counts and bleeding events. This promotes the need for long-term individualized monitoring, preventative strategies, and treatment options. Clinical practice should be guided not only with the intent to increase the platelet count to a level that decreases the risk of bleeding but also to improve the patient's quality of life while minimizing risks with associated comorbidities.
